# Mechanisms of APOBEC3 Packaging into HIV-1

**DOI:** 10.3390/v18030389

**Published:** 2026-03-20

**Authors:** Mirriam Nzivo, Christoph G. W. Gertzen, Tom Luedde, Holger Gohlke, Carsten Münk

**Affiliations:** 1 Clinic of Gastroenterology, Hepatology and Infectious Diseases, Medical Faculty, Heinrich Heine University Düsseldorf, 40225 Duesseldorf, Germany; mirriam.nzivo@med.uni-duesseldorf.de (M.N.); tom.luedde@med.uni-duesseldorf.de (T.L.); 2 School of Biological Sciences, Jomo Kenyatta University of Agriculture and Technology, Nairobi P.O. Box 62000-00200, Kenya; 3 Institute for Pharmaceutical and Medicinal Chemistry, Heinrich Heine University Düsseldorf, 40225 Duesseldorf, Germany; christoph.gertzen@hhu.de (C.G.W.G.); gohlke@uni-duesseldorf.de (H.G.); 4 Center for Structural Studies, Heinrich Heine University Düsseldorf, 40225 Düsseldorf, Germany; 5 Institute of Bio- and Geosciences (IBG-4: Bioinformatics), Forschungszentrum Jülich GmbH, 52425 Jülich, Germany

**Keywords:** APOBEC3s, HIV-1, packaging, GAG, RNAs

## Abstract

Apolipoprotein B mRNA editing enzyme catalytic polypeptide 3s (APOBEC3s, A3s) are single-stranded DNA cytidine deaminases with antiviral activity against diverse DNA and RNA viruses. The human APOBEC3 locus encodes seven members: A3A, A3B, A3C, A3D, A3F, A3G, and A3H. Of these, A3C, A3D, A3F, A3G, and A3H are packaged into HIV-1, lacking the viral infectivity factor (VIF, HIV-1Δ*vif*), while A3D, A3F, A3G, and A3H hap II exhibit strong antiviral activity. Packaging of A3s into virions is critical for viral restriction, yet the underlying mechanisms remain incompletely understood. A3 incorporation requires interactions with the GAG polyprotein, especially the matrix (MA) and nucleocapsid (NC) domains, and binding to cellular or viral RNAs. Specific amino acid residues within A3 proteins mediate these contacts, and A3G localization to lipid rafts facilitates packaging. While A3F and A3G incorporation have been extensively characterized, mechanisms for other A3s remain poorly defined. This review synthesizes current knowledge on A3 packaging, emphasizing the interplay of protein, RNA, and membrane determinants in efficient virion incorporation.

## 1. Introduction

Human APOBEC3s (A3s) are single-stranded polynucleotide cytidine deaminases arranged in tandem on chromosome 22 [1,2]. The expansion of the *A3* family and their positive selection during evolution in placental mammals indicate that A3 proteins play a fundamental role in host defense [1,2]. The number of *A3* genes varies among species, ranging from a single gene in mice and rats to 18 in bats [1,2,3]. Humans and other primates express seven A3 proteins. Four of these A3B, A3D, A3F, and A3G contain two zinc-coordinating cytidine deamination (CD) domains, while A3A, A3C, and A3H contain a single CD domain. These domains are classified into three subgroups: Z1 (A3A and the C-terminal CD of A3B and A3G), Z2 (A3C, the N-terminal CD of A3B and A3G, and both domains of A3D and A3F), and Z3 (A3H) [1,4]. It is believed that two-domain A3s arose through the duplication and recombination of ancestral single-domain A3s [1,2].

To restrict HIV-1 replication, A3 proteins are packaged into assembling viral particles. However, HIV-1 expresses the accessory viral infectivity factor (VIF), which counteracts A3 antiviral activity. VIF recruits an E3 ubiquitin ligase complex composed of cullin 5, elongin B/C, RING-box protein 2, core binding factor β, and Ariadne homolog 2, mediating A3s ubiquitination and subsequent proteasomal degradation [5,6]. In addition to promoting degradation, VIF inhibits A3 proteins through degradation-independent mechanisms, all of which interfere with packaging [7,8]. For instance, VIF inhibits translation of A3G mRNA, reducing A3G cellular levels and hence incorporation [9,10,11]. VIF binds to the 5′UTR of A3G RNA, resulting in ribosome stalling, inhibiting A3G mRNA translation [9,12]. VIF blocks A3G transcription by competing with RUNX for CBF-β binding [12]. A3G and VIF share the same binding domains in viral RNA. By competing for the same sites and blocking such domains, VIF reduces A3G packaging [12]. VIF intercepts A3G bound to genomic RNA to exclude A3G from the complex, reducing packaging [13]. A3s restrict viruses via cytidine deamination-dependent and independent mechanisms, such as inhibiting reverse transcription by blocking or directly binding to RT and inhibiting integration [14,15,16], and can target both ssDNA and ssRNA [14,15,16,17]. Although A3B and A3C exhibit limited activity against HIV-1, they effectively restrict simian immunodeficiency virus (SIV) [18,19,20]. In contrast, A3D, A3F, A3G, and A3H hap II are potent anti-HIV restriction factors. A3A is not packaged into HIV-1 particles and shows no anti-HIV-1 activity [21,22,23,24].

The mechanism of A3 packaging has been extensively investigated for A3G. The N-terminal domain (NTD) of A3G mediates packaging, while the C-terminal domain (CTD) carries the catalytic activity [22,25,26]. Additionally, the NTD enhances the deamination activity of full-length (FL) A3G by increasing substrate affinity [27,28]. In contrast to A3G, both the NTD and the CTD of A3F contribute to packaging [29,30,31,32]. Some studies report that the A3F CTD is comparably packaged to full-length A3F, while others observe reduced packaging efficiency [29,32,33]. Although mutants lacking the A3F NTD or containing only the CTD can still be packaged, they exhibit diminished antiviral restriction compared to full-length A3F, indicating that the NTD facilitates deamination by enhancing substrate binding [29,30,31,32,33].

Across the studies evaluated, most of which are based on A3 overexpression, a consensus has emerged that A3 proteins interact with the HIV-1 group-specific antigen (GAG) polyprotein, either directly or through an RNA bridge. Both viral and cellular RNAs participate in packaging. Specific residues within A3 proteins contribute to packaging by mediating interactions with GAG or RNA. The role of A3 multimerization in packaging remains unresolved. Although many A3s are localized to P-bodies, this localization does not appear to influence packaging. Conversely, association with lipid rafts correlates with efficient A3 incorporation into HIV-1 particles.

## 2. APOBEC3s Residues Important for Packaging into HIV-1

Restriction of HIV-1 by A3 proteins requires their recognition of viral particles prior to budding. Most studies support a model in which A3 proteins are selectively packaged into virions rather than passively diffusing into them. Therefore, A3 proteins must possess molecular features that sense and bind assembling HIV-1 particles. Research aimed at defining this sensing interface has primarily focused on A3G.

**Figure 1:**
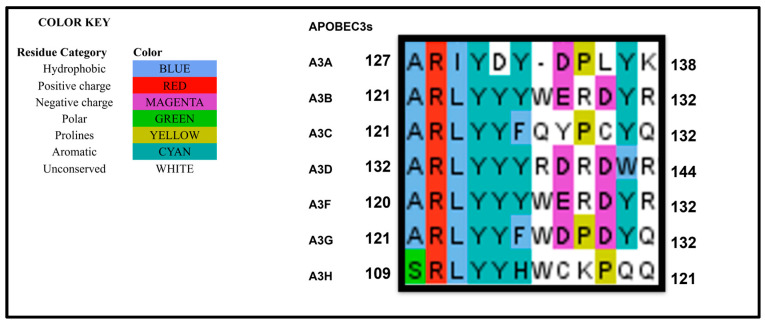
An alignment of the human APOBEC3s RLYY(F/Y)W motif. Single-domain A3s were aligned with the N-terminus of the two-domain A3s. The region RLYY(F/Y)W was conserved among the A3s. The region was found to be important for virus packaging of A3 proteins.

**Figure 2:**
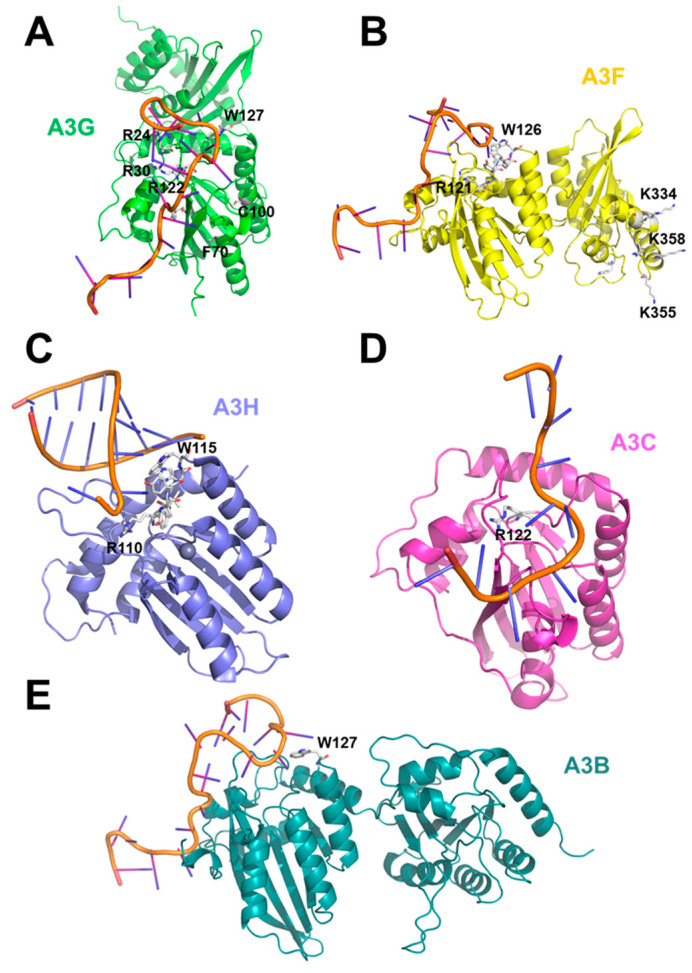
Structures of different APOBEC3s (A3s) with (modeled) nucleotides bound. Structures of (**A**) APOBEC3G (A3G) (green, AlphaFold model), (**B**) APOBEC3F (A3F) (yellow, AlphaFold model), (**C**) APOBEC3H (navy, PDB ID: 8FVI [52]), (**D**) APOBEC3C (A3C) (magenta, PDB ID: 3VOW [53]), and (**E**) APOBEC3B (A3B) (forest green, AlphaFold model) are shown with residues important for packaging/nucleotide binding highlighted as white sticks. (**A**) The nucleotide-bound structure was made by aligning the model to the RNA-bound structure of A3G (PDB ID: 8J62 [54]); some residues that are important for packaging, e.g., F70, are in the hydrophobic core and likely contribute by facilitating correct folding rather than RNA binding. (**B**) The nucleotide-bound structure of A3F was made by aligning the model to the RNA-bound structure of A3G (PDB-ID: 8J62 [54]). A3F likely has two distinct sites that could bind (D/R)NA: residues R121-W126 and residues K334, K355, and K358. (**C**) The cryo-EM structure of A3H was solved with nucleotides. (**D**) The A3C structure was made as in Ref. [55]. (**E**) The nucleotide-bound A3B structure was made by aligning the model to the RNA-bound structure of A3G (PDB-ID: 8J62 [54]). The locations of the highlighted residues suggest that the APOBECs are mainly packaged into viral particles via interactions with (D/R)NA. Generally, complete crystal structures were prioritized over AlphaFold models. With 8CX0, a complete structure for A3G is also available [13].

Early studies demonstrated that the N-terminal region of A3G (amino acid residues 1–196) is essential for its incorporation into HIV-1ΔVIF particles [22,34]. This region also contributes to RNA binding [35,36]. Although mass spectrophotometry analysis suggested that the C-terminal catalytic domain of A3G can bind RNA [37], other biophysical approaches, including gel-shift assays and rotational anisotropy, did not detect nucleic acid binding by A3G-CD2 [28,33]. Mutational analysis of A3G residues within the N-terminal catalytic domain (CD1) identified several key residues R24, R30, F70, Y91, C100, R122, and W127, that are critical for both virion incorporation and RNA binding [34,35,38,39,40,41]. Substitutions at R122 and W127 alter both charge and protein structure [39]. These residues lie within the RLYY(Y/F/H)W motif (A3G AA 121–132), a conserved RNA-binding motif shared among A3G, A3F, and all A3H haplotypes that mediates virion packaging [42,43]. A sequence alignment shows that the motif is also conserved across all A3s (Figure 1. Some of the residues that were identified to be critical for viral packaging (e.g., F70, C100, Y91) are located at the hydrophobic core of the protein and are likely to be relevant for the correct folding of A3G rather than for interactions that facilitate packaging. The residues involved in A3G packaging have been illustrated in Figure 2A.

In A3F, mutation W126A within the RLYY(Y/F/H)W motif (in A3F AA 121–126) reduces packaging efficiency by ~70% compared to wild-type A3F, likely due to reduced interaction with 7SL RNA, although binding to genomic RNA and 5S RNA is unaffected [44]. Another packaging determinant for A3F is the linker region between its two catalytic domains (104–156) [45]. Replacement of this linker with that from rhesus A3G decreased A3G packaging [32]. This linker connects interaction with HIV-1 nucleocapsid (NC), mediating packaging, and its interaction persists after RNAse treatment, indicating direct protein–protein interaction [45,46]. Similar linkers in A3G and murine A3 also contribute to packaging [40,42,45,46]. Beyond the linker region, residues in the A3F C-terminal catalytic domain modulate nucleic acid binding [31,33]. Mutation W310A impairs nucleic acid binding [31], and a distal positively charged patch comprising K334, K337, K352, K355, and K358 engages ssDNA [33]. A3F seems to have two different sites that could bind (D/R)NA: residues R121-W126 and residues K334, K355, and K358. Figure 2B shows A3F residues involved in nucleic acid binding.

In A3H hapII, the RLYY(Y/F/H)W motif (amino acids 110–115) is also critical for packaging, supporting interaction with 7SL RNA (Figure 2C) [43]. Mutation W115A abolishes RNA binding and prevents packaging. A3H haplotypes differ by non-synonymous single nucleotide polymorphisms at positions 105R/G, 121K/D, and 178E/D as well as by codon deletion at position 15 (Δ15) [43,47]. A3H hap I encodes GKE at these positions, while hap II has RDD [43,47]. Residue 105, located near the conserved motif, determines packaging efficiency by modulating GAG interaction through enhanced RNA binding [43,47]. Although A3H hap I GKE is poorly expressed, it interacts with the MA-CA domain, whereas hap II RDD interacts with NC in an RNA-dependent manner [47]. A3H hap II exhibits lower binding to host small RNAs, resulting in reduced virion incorporation [43]. The Δ15 deletion in hap III and hap IV prevents RNA binding and abolishes virion packaging; deletion of N15 in hap II has a similar effect [43].

In A3C, mutation R122A, located near a nucleic acid binding site, disrupts RNA binding, particularly to 5.8S RNA, and blocks packaging (Figure 2D) [48]. In A3B, W127 is important in single-stranded DNA binding (Figure 2E) [49].

To visualize these determinants, we mapped key residues onto crystal structures of multiple A3s (Figure 2A–E). Most packaging-related residues cluster around beta sheet 4 and its adjacent loop within the N-terminal catalytic domain. Additional residues are located at other structural features: in A3G, R24 lies in loop 2, R30 in beta-sheet 1, H65 and F70 in alpha-helix 2, Y91 in beta-sheet 3, W94 in the turn adjacent to beta-sheet 3, and C100 in alpha-helix 3. In A3H, N15 is positioned in loop 2. In A3F, W310 lies in beta-sheet 4 of the C-terminal domain. A positively charged surface patch in A3F (K334, K337, K352, K355, K358) also mediates ssDNA binding [33]. Additionally, Y315 in A3G has been shown to interact with RNA and ssDNA [50], although this interaction has not been investigated in the context of HIV-1 packaging.

Overall, the residues that were identified in A3s to facilitate viral packaging (Table 1) are all at sites that likely influence correct folding, directly bind (D/R)NA, or, in the case of A3F, are located at a second site that could bind (D/R)NA. None of these residues are in positions that could contribute to the binding of other A3s. Although A3F has a second site, the cluster of lysines present hints at nucleotide binding (phosphate backbone), as these are not residues that are prominent in protein–protein interactions [51]. Hence, viral packaging of A3s apparently is not dependent on the formation of dimers or higher-order oligomers in the absence of (D/R)NA.

**Table 1:** Residues in APOBEC3s that are important for packaging.

APOBEC3s	Amino Acid Residues	References
A3B	W127	[49,54]
A3C	R122	[48,53]
A3D		
A3F	Linker space between the two cytidine deaminases (aa 104–156)	[30,31,33,44,45]
	RLYY(F/Y)W 121–126	
	CD 2 loop 7 W310	
	K334, K337, K352, K355, K358	
A3G	Linker space between the two cytidine deaminases (aa 104–156)	[35,37,38,39,40,41,42,50,54,56,57]
	R24	
	R30	
	H65	
	F70	
	Y91	
	W94	
	C97	
	C100	
	RLYY(F/Y)W 122–127	
	Y315	
A3H	N15, 110 RLYYHW115	[43,47,52]
	G105	

## 3. GAG and APOBEC3s Interaction

**Figure 3:**
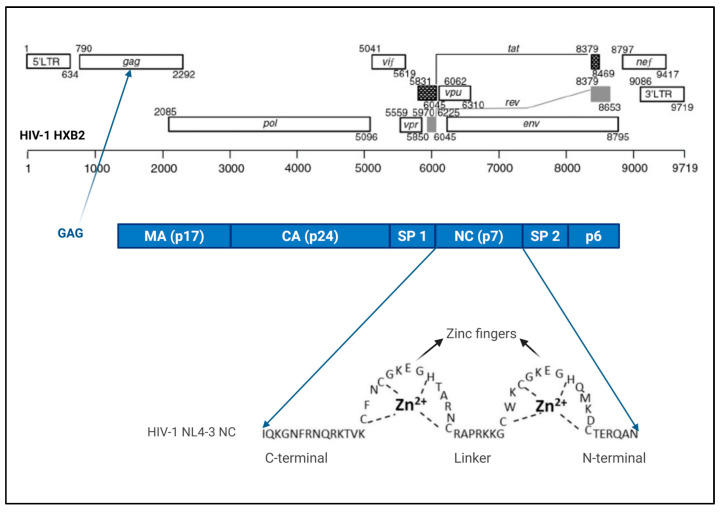
The full sequence of the HIV-1 genome was adapted from Grossman et al. [77]. Shown are the protein domains that are generated after protease cleavage of GAG polyprotein: matrix (p17), capsid (p24), nucleocapsid (NC, p7), SPI, SP2 and p6. The majority of A3s have been reported to interact with NC. Matrix has been reported to interact with A3C and A3H, allowing their packaging.

The GAG of HIV-1 is a 55 kDa polyprotein that represents the major structural driver of viral assembly. It ensures efficient packaging of the viral genomic RNA and coordinates interactions with other viral and host factors to generate particles containing all components required for productive infection. Following proteolytic cleavage, the GAG polyprotein yields matrix (MA), capsid (CA), spacer 1, nucleocapsid (NC), spacer 2, and p6 (Figure 3) [58,59,60]. Among these, the NC domain is highly basic in nature and contains two zinc finger motifs that mediate binding and chaperone functions for nucleic acids. These zinc fingers provide hydrophobic interfaces that promote nucleic acid interactions [61,62,63,64]. Owing to its structural plasticity, NC exhibits broad RNA-binding capacity and interacts with multiple cellular and viral RNAs [58,65]. In addition to NC, the MA domain also interacts with RNA through a highly N-terminal region spanning amino acids 17 and 32 [58], and interactions are largely biased toward tRNAs [66].

Importantly, GAG-RNA interactions undergo dynamic changes during virion assembly [61,63,66]. In the cytoplasm, GAG preferentially associates with G/U-rich RNA sequences [63,66]. During assembly, this preference shifts toward A-rich RNA sequences, facilitating selective recruitment of the viral genomic RNA. Following proteolytic cleavage of GAG, NC-binding specificity reverts back to G/U-rich RNAs [63,66].

In virus-producing cells, GAG interactions with A3C, A3F, A3G, and A3H are predominantly reported to be RNA-dependent [26,35,44,45,47,61,67,68,69,70,71,72,73,74]. However, some studies also report direct RNA-independent binding between GAG and A3G [46,75,76]. A3G, A3F, and A3H hap II interact with NC in an RNA-dependent manner [45,47,61,75], whereas A3H hap I predominantly binds the CA-MA region [47]. A3C interacts with MA in a similarly RNA-dependent fashion [72], and comparable behavior was observed with A3C binding to simian immunodeficiency virus (SIV) NC [48].

In support of RNA-independent interactions, Alce and Popik demonstrated that NC alone, expressed as a glutathione-S-transferase (GST) fusion, was capable of binding A3G in vitro and that both zinc fingers contribute to A3G packaging; zinc finger mutations greatly reduced packaging efficiency [75]. Additionally, two studies reported successful coimmunoprecipitation of GAG and A3G after RNAse treatment [46,76]. These discrepancies may reflect the presence of either direct A3G-GAG interactions or context-dependent differences in experimental conditions. Cen et al. and Douaisi et al. proposed an alternative explanation involving a small RNA bridge potentially shielded by A3G and GAG/NC, rendering it resistant to RNAse digestion [46,76].

## 4. RNAs and APOBEC3s Packaging

A3F, A3G, and A3H have been shown to bind HIV-1 GAG or NC through RNA-dependent interactions, as demonstrated by crosslinking immunoprecipitation combined with deep sequencing approaches [61,68,73,74]. Despite these advances, the specific RNA required for binding A3 proteins to NC remains debated, with viral genomic RNA, non-coding (Y RNAs, Alu, U RNAs), and coding cellular RNAs all being proposed as potential mediators. Several studies suggested that the viral genomic RNA plays a role in A3G packaging [70,78,79]. However, the majority of reports indicate that viral genomic RNA is not essential for the packaging of A3C, A3F, A3G, or A3H [34,35,44,46,61,71,80]. Supporting a minor contribution of viral RNA, Becker et al. showed that the presence of genomic RNA increases A3G incorporation by approximately 30% relative to GAG virus-like particles lacking viral RNA [81].

Among cellular RNAs, 7SL RNA has been most extensively investigated due to its unusually high abundance in HIV-1 virions, where it is present at levels sevenfold higher than the viral genomic RNA [82,83]. 7SL RNA, the RNA component of the signal recognition particle (SRP), is a 300-nucleotide non-coding, highly structured RNA involved in co-translational protein targeting to the endoplasmic reticulum [84,85]. Multiple studies have linked A3 packaging to interactions with 7SL RNA [42,43,44,48,70,72,78,79,86,87]. Three reports further proposed that only A3 proteins with anti-HIV-1 activity A3F, A3G, and A3H hap ll specifically interact with 7SL RNA, mediated through the conserved A3 RLYY(F/Y)W motif [42,43,72]. However, this motif is present in all human A3s as mentioned earlier (Figure 1), challenging its specificity. Consistent with this, Stauch et al. observed that A3C binds 7SL but that such binding is not required for its incorporation into virions [48]. Instead, their work indicated that interaction with 5.8S RNA is critical for A3C packaging [48]. In contrast, Apolonia et al. reported no detectable 7SL binding by A3F [61]. Notably, 7SL RNA itself may be packaged into HIV-1 via direct interaction with GAG or NC [88,89]. However, a study by Onafuwa-Nuga reported that 7SL RNA was packaged independently of viral determinants for RNA packaging. In that study 7SL was packaged in GAG deletion mutants lacking NC, MA, parts of CA, and the late domain [82]. Therefore, the mechanism underlying the selective recognition of RNA by A3s remains unresolved.

It was also proposed that A3G, A3F, and A3H promiscuously bind to a broad array of cellular and viral RNAs to guarantee incorporation into virions [61,68]. However, A3 proteins demonstrate a preference for viral RNAs over cellular RNAs [61,74], raising the question of how this selective bias is achieved. York et al., using cross-linking immunoprecipitation and deep sequencing, demonstrated that A3F, A3G, and A3H do not bind RNAs randomly. Instead, similar to NC, these A3 proteins prefer RNAs enriched in guanosine (G) and adenine (A) and, importantly, RNAs that are not translated by ribosomes [74]. This preference for G- and A-rich RNA sequences by A3G had also been reported earlier [67]. York et al. concluded that A3 proteins mimic NC in their RNA-binding specificity [74]. More recently, it was reported that human and rhesus A3G preferentially bind RNA AA dinucleotides using residues (Y124-W127) that are important for viral packaging of A3G [13,52,54,73], a finding paralleled for A3F by Pacheco et al., who demonstrated that both A3F domains recognize AA motifs [32]. This contrasts with NC, which primarily binds unpaired guanosines [74]. It was concluded that sequence-specific RNA recognition is crucial for A3G packaging [13,73]. Because viral RNAs are highly enriched in adenine [57,73,74], A3 proteins preferentially associate with viral sequences. Accordingly, A3 incorporation may be enabled by binding to any accessible RNA that fits these sequence features (Table 2). The structure of A3H revealed that an A3H dimer forms around a short RNA duplex [90,91]. The property of A3H to bind RNA duplexes may facilitate its virion incorporation [90].

## 5. A3-Multimerization and A3s Packaging

Many A3 proteins self-associate to form dimers or higher-order oligomers. Structural studies have detected A3A, A3B, A3H, and A3G as dimers, whereas A3C exists as a monomer [24,36,53,90,92]. RNA binding has been closely linked to multimerization of several A3 proteins [24,69,93,94]. Accordingly, A3G oligomerization is sensitive to RNAse treatment, suggesting that RNA scaffolds facilitate or stabilize multimer formation [38]. Each A3 protein appears to employ a distinct mode of dimerization or oligomerization, which has been proposed to contribute to diverse functional outcomes, including packaging into virions, subcellular localization, and catalytic activity [24,36,95].

Whether multimerization is required for efficient A3 incorporation into HIV-1 remains controversial [24,38,69,78]. Burnett and Spearman used fluorescence resonance energy transfer (FRET) to show that A3G-RNA multimers are selectively recruited to virus-like particles [69]. Using fluorescence fluctuation spectroscopy (FFS), Li et al. further concluded that A3 proteins that form multimers are packaged more efficiently into virions [24]. In contrast, Khan et al. reported that A3G is incorporated in a monomeric state and that both packaging-competent and packaging-defective A3G variants retain the ability to oligomerize [78]. Additionally, the A3G multimerization mutant C97A was reported to be packaged into virions [38]. However, Li et al. argued that A3G-C97A is poorly expressed and fails to package in HIV-1Δ*vif* [24]. These conflicting observations suggest that multimerization may enhance packaging efficiency for some A3 proteins but is unlikely to constitute a universal or primary determinant of A3 incorporation into virions. Instead, multimerization may play a more prominent role in regulating A3 catalytic activity rather than directly controlling packaging.

## 6. Subcellular Localization of A3s and Packaging

**Figure 4:**
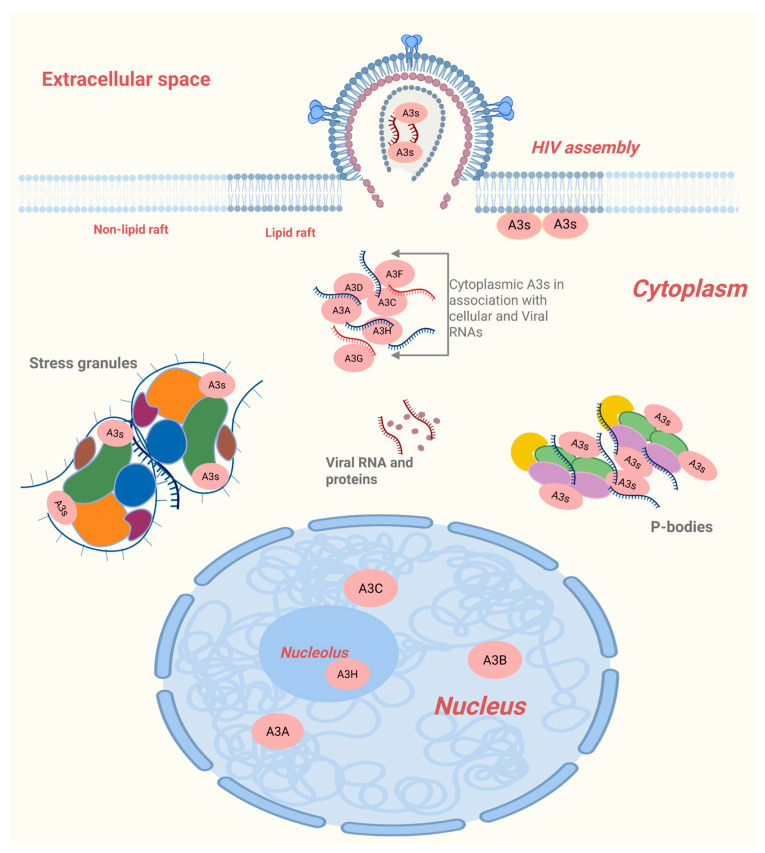
APOBEC3s localization in the cell. Some A3s appear in the cytoplasm, while others in the nucleus. A3H is found in the nucleolus. A3s can be sequestered in RNA-rich domains such as stress granules and P-bodies. Some A3s also localize at lipid rafts, where they are suspected to interact with GAG.

In the cell, A3s can be found in the cytoplasm or the nucleus and/or in association with stress granules, lipid rafts and P-bodies, all of which have been investigated in relation to packaging (Figure 4).

APOBEC3s are uniquely distributed in cells. A3G, A3F and A3D are localized in the cytoplasm and are all well packaged in HIV-1 [96]. A3G residues within amino acids 1 to 60 and amino acids 113 to 128 cooperate to determine APOBEC3G’s cytoplasmic localization [96]. A3C and A3A are distributed in the nucleus and the cytoplasm, but only A3C is packaged [20]. A3B is restricted in the nucleus and was reported to be well packaged in HIV-1 [20,97]. Amino acid residues 18, 19, 22, and 24 in A3B were the determinants for nuclear versus cytoplasmic localization [20]. A3H localizes in the cytoplasm and the nucleolus, and distinct residues are responsible for its localization in the different compartments [98]. Residues in loop 1 and loop 7 or α-helix 6 residues together with duplex RNA are responsible for retaining A3H in the cytoplasm, while select residues in loop 1 and loop 3 retain A3H in the nucleolus [98,99]. HIV-1 GAG assembles at the plasma membrane, and therefore A3s located in the cytoplasm can easily interact with GAG mainly through RNA-dependent and direct protein interaction. GAG and A3s have also been located at the point of GAG assembly, the lipid rafts.

Lipid rafts are organized microdomains of the plasma membrane enriched in cholesterol and sphingolipids. Newly synthesized A3G has been observed to associate with these domains, and HIV-1 GAG similarly accumulates at lipid rafts during virion assembly [45,56,78]. This spatial overlap supports the idea that lipid rafts serve as the point of interaction between A3 proteins and GAG. The link between lipid rafts and A3G incorporation is further strengthened by the observation that the packaging-defective A3G mutant, W127A, fails to associate with lipid rafts [78]. Ma et al. demonstrated that the lipid raft-associated low molecular mass (LMM) A3G complex, rather than the high molecular mass complex form, is the principal source of A3G incorporated into virions [56]. They further showed that residues 105–156 in the A3G N-terminal domain mediate its lipid raft localization [56]. Because HIV-1 GAG concentrates at lipid rafts during virion assembly [100,101], the convergence of Gag and A3G at these membrane domains strongly suggests that lipid rafts facilitate A3G-Gag interactions required for viral packaging. In the cytoplasm, A3s also co-localize with RNA-rich domains such as P-bodies and stress granules.

Processing bodies (P-bodies) are cytoplasmic ribonucleoprotein granules involved in mRNA translational repression, storage, and decay, and they contain key RNA silencing factors such as Argonaute proteins (Figure 4) [102]. Early interest in the potential role of P-bodies in A3 protein packaging stemmed from observations that many A3 proteins, including A3B, A3C, A3D, A3F, A3G, and A3H, localized to P-bodies [44,103,104,105,106,107,108]. This hypothesis was further supported by initial reports suggesting that HIV-1 RNA also localizes to these granules [109,110] and that HIV-1 interacts with proteins associated with P-bodies and miRNA pathways. However, subsequent studies demonstrated that neither HIV-1 GAG nor genomic RNA is associated with P-bodies [104,105], challenging earlier assumptions. Functional studies have consistently shown that A3 localization in P-bodies does not determine viral incorporation. Although Phalora et al. reported a correlation between A3 protein accumulation in P bodies and their incorporation into virions, depletion of P-bodies using siRNA and shRNA did not impair A3s packaging [105]. Likewise, Wang, Tian et al. demonstrated that A3F remains efficiently packaged even when P-bodies are disrupted with actinomycin D [44]. Izumi et al. further confirmed that neither A3G nor A3F incorporation into HIV-1 virions depends on P-body localization [104]. These findings support a model in which P-bodies act as sequestration sites that limit the availability of A3 proteins to prevent unintended mutagenesis of host DNA, rather than participating in packaging [61,104]. Consistent with this, only newly synthesized A3G, and not A3G contained in high molecular mass ribonucleoprotein complexes, is efficiently packaged into virions [56,106]. Thus, while many A3 proteins localize to P-bodies, this localization does not influence their incorporation into HIV-1 particles or antiviral function. Like P-bodies, SGs are sequestration points for A3s and do not influence packaging. A3G and A3B have been observed to localize in stress granules [103,111,112]. While lipid rafts serve as a point of interaction between A3s and GAG, it is important to note that A3G has been reported to interact with viral RNA immediately after nuclear export [81]. This does not refute interaction with GAG at lipid rafts since, for packaging to occur both A3G and viral RNA need the nucleocapsid.

## 7. APOBEC3s Packaging in Other Viruses

A3s mutational footprints have been detected across several virus families, where they contribute either to antiviral restriction or to viral evolution [14,113]. Among retroviruses, broad A3 packaging has been documented; whether A3 packaging into other viruses is a requirement for antiviral activity is less well understood. In human T-cell leukemia virus type 1 (HTLV-1), A3A, A3B, A3C, A3D, A3F, A3G, and A3H hap II are all packaged into nascent virions [114,115]. A3G has been shown to colocalize with murine leukemia virus (MLV) GAG particles in a viral RNA genome-independent manner [76,116]. In these studies, RNAse treatment did not interfere with GAG-A3G coimmunoprecipitation [76,116]. Whether in these systems non-viral RNAs mediated the A3 encapsidation is unknown.

In the hepatitis B virus (HBV), several A3 members A3A, A3B, A3C, A3F, and A3G, interact with the core proteins [117,118,119,120,121]. Packaging of A3G in HBV requires viral reverse transcriptase (RT) and the ε (epsilon) RNA encapsidation signal, which together facilitate A3G–core interaction [118,122]. In contrast to RNA-mediated mechanisms, Zhao et al. observed direct A3G–core binding without the need for viral or cellular RNAs [123], suggesting that the A3G–protein interfaces may differ between retroviruses and hepadnaviruses.

Outside of retroviruses and HBV, A3 interactions have been found in other virus families. In human papillomavirus (HPV), A3C, but not A3A, interacts with the major capsid protein L1 [124], indicating selective A3 recruitment. Also, in RNA viruses that do not reverse transcribe, such as coronaviruses, host mutational signatures attributed to A3 activity are prevalent [125,126,127] and A3C, A3F, and A3H were shown to bind the coronavirus nucleoprotein, leading to viral inhibition [128]. A3A mutations have been observed in the SARS-CoV-2 genome [17]. This was interesting, as A3’s substrate is known to be ssDNA. Nakata and colleagues observed that A3A caused C-U editing at hairpin regions. Based on the current model, A3A recognizes nucleotides with a C2′-endo sugar pucker, which is typical for ssDNA and can also occur at the tips of RNA hairpins [17]. Moreover, a strong A3-associated mutation pattern has been observed in the monkeypox virus (MPOX) genome [129,130,131,132,133]. Notably, recent MPOX outbreaks have been linked to A3-driven mutational acceleration, potentially enhancing viral diversification [129,130,131,132,133]. However, unlike enveloped RNA viruses, the mechanism by which cytoplasmic poxviruses interact with A3 proteins remains unresolved [134].

Although the packaging of A3s by viruses other than HIV has not been extensively studied, the diverse strategies viruses have evolved to evade A3 restriction underscore the strong selective pressure these enzymes exert on viral replication and evolution. Similar to HIV-1, several complex retroviruses, including HIV-2, simian immunodeficiency viruses (SIVs), and feline immunodeficiency virus (FIV), encode VIF, which blocks A3 packaging by targeting A3 proteins for degradation [135]. Human T-cell leukemia virus type 1 (HTLV-1) nucleocapsid (NC) protein inhibits A3G incorporation into virions [136]. Mutations in HTLV-I NC relieve this inhibition, increasing A3G packaging and subsequent restriction [136].

In addition, deltaretrovirus antisense proteins were found to play some role in A3 counteraction. The HTLV-2 antisense protein APH-2 inhibits the deaminase activity of both human and simian A3G, while the HTLV-1 bZIP factor (HBZ) and simian T-cell leukemia virus type 1 (STLV-1) bZIP factor (SBZ) potently inhibit simian A3G deaminase activity and only weakly inhibit human A3G [137].

Murine leukemia virus (MLV) has evolved multiple mechanisms to counteract mouse A3 (mA3). Viral RNA interferes with the interaction between mA3 and GAG, thereby excluding mA3 from virions, and the viral protease cleaves mA3 after virion maturation [138]. In addition, the MLV-encoded p50 protein interacts with mA3 and prevents its packaging [139]. MLV also encodes a glycosylated form of GAG that stabilizes the virion core and restricts mA3 access to the reverse transcription complex [140]. Mouse mammary tumor virus (MMTV) counteracts mouse A3 by increasing the rate of reverse transcription [141]. The MMTV Rem protein antagonizes the A3-related activation-induced cytidine deaminase (AID) and may influence mutations by mA3 [142,143]. Russell and colleagues reported that the prototype foamy virus BET inhibited human A3G and A3F packaging [144]. The BET protein impedes human A3G and A3C by abolishing their dimerization and sequestering them in insoluble complexes, inhibiting their packaging [145,146]. Epstein–Barr virus (EBV) and Kaposi’s sarcoma-associated herpesvirus (KSHV) inhibit A3 activity through the large subunit of ribonucleotide reductase, EBV BORF2 and KSHV ORF61, respectively, which bind A3A and A3B, inhibit their deaminase activity, and relocalize them from the nucleus to the cytoplasm [147].

In addition to active antagonism, some viruses evade A3-mediated restriction through passive escape by depleting A3-preferred target motifs (5TC) from their genomes. This underrepresentation, termed the “A3 evolutionary footprint”, reflects long-term selective pressure exerted by A3 enzymes. Evidence of A3 selection pressure has been reported in 22% of annotated human viruses [148]. Papillomaviridae and Polyomaviridae exhibit extensive, genome-wide A3 footprints, indicative of strong selection pressure, whereas Gammaherpesviridae and Adenoviridae display A3 footprints primarily localized to lytic origins of replication [148]. In herpes simplex virus 1 (HSV-1), A3 hotspot motifs are more frequent in immediate early and early genes than in late genes [149]. Consistently, TC dinucleotides are the preferred targets of several A3 enzymes which are depleted in human papillomaviruses and BK polyomavirus [149,150]. Endemic coronaviruses (HCoV-NL63, HCoV-229E, HCoV-HKU1, HCoV-OC43) display pronounced evolutionary A3 footprints, in contrast to zoonotic coronaviruses including SARS-CoV-2, SARS-CoV-1, and MERS-CoV [148].

## 8. Outlook and Open Questions

The importance of A3 proteins in antiviral immunity cannot be overstated. Their broad activity against diverse viral families makes them pivotal targets for the development of novel antiviral strategies. Because packaging into viral particles is a prerequisite for the mutagenic activity of many A3 enzymes, elucidating the molecular mechanisms that govern A3 incorporation into virions remains critically important.

A major conclusion from current research is that A3 packaging is regulated by multiple determinants rather than a single universal mechanism. For retroviruses such as HIV-1, the GAG polyprotein is indispensable for A3 incorporation. GAG serves as a precursor to several structural components of the virion, and different A3 proteins may therefore interact with distinct GAG domains. Whether these interactions occur directly through protein–protein contacts or indirectly through RNA scaffolding appears to vary among A3 family members.

Evidence indicates that RNA frequently plays a central role in mediating A3-GAG interactions, either as a bridge facilitating binding or as a determinant of A3 selective incorporation. Several A3 enzymes possess amino acid residues known to bind RNA directly, and for A3G, extensive work has demonstrated an RNA-dependent interaction with GAG. However, for most A3 proteins, the precise molecular interfaces and contributions of both viral and cellular RNAs remain poorly defined.

While A3G packaging has been extensively characterized, and recent studies broadly agree on a mechanistic framework, the same cannot be said for other A3 enzymes. For A3A, A3B, A3C, A3D, A3F and A3H, published data remain inconsistent or too limited to draw definitive conclusions, highlighting a significant knowledge gap. This lack of clarity limits our understanding not only of how these enzymes restrict viruses, but also of how they could be intentionally exploited to drive antiviral mutation or viral attenuation.

Given their natural ability to edit and inhibit viral genomes, A3 proteins represent promising candidates for next-generation antiviral interventions. A deeper mechanistic understanding of their packaging, spanning GAG interactions, RNA dependencies, and cell-specific regulation, will be essential for harnessing their full therapeutic potential. Continued research of the selective incorporation of A3 proteins into diverse viruses could pave the way for innovative approaches to both viral treatment and prevention.

## Figures and Tables

**Table 2:** RNAs that interact with A3s.

Viral RNA
7SL RNA
Y RNAs
Alu RNAs
Cellular mRNAs
G and A-rich RNAs
A rich RNA
AA dinucleotides
5.8s RNA
U RNAs

## Data Availability

No new data were created or analyzed in this study.

## Abbreviations

A3s	APOBEC3s
AID	Activation-induced cytidine deaminase
APOBEC3s	Apolipoprotein B mRNA editing enzyme catalytic polypeptide 3s
HIV-1	Human immunodeficiency virus
CA	Capsid
CD	Cytidine deamination
CTD	C-terminal domain
DNA	Deoxyribonucleic acid
EBV	Epstein–Barr virus
FIV	Feline immunodeficiency virus
FFS	Fluorescence fluctuation spectroscopy
FRET	Fluorescence resonance energy transfer
GAG	Group-specific antigen
GST	Glutathione-S-transferase
HBV	Hepatitis B virus
HBZ	HTLV-1 bZIP factor
HCov	Human coronavirus
HTLV	Human T-cell leukemia virus
HPV	Human papillomavirus
HSV	Herpes simplex virus
KSHV	Kaposi’s sarcoma-associated herpesvirus
LMM	Low molecular mass
mA3	Mouse A3
MA	Matrix
MERS-CoV	Middle East respiratory syndrome (MERS) CoV
MLV	Murine leukemia virus
MMTV	Mouse mammary tumor virus
MPOX	Monkeypox virus
NC	Nucleocapsid
NTD	N-terminal domain
P6	Protein 6
RNA	Ribonucleic acid
RT	Reverse transcriptase
SARS-CoV	Severe acute respiratory syndrome coronavirus 2
SIV	Simian immunodeficiency virus
SP 1	Spacer protein 1
SP 2	Spacer protein 2
STLV	Simian T-cell leukemia virus type
VIF	Viral infectivity factor
